# Robust 3D Bloch‐Siegert based $B_{1}^{+}$ mapping using multi‐echo general linear modeling

**DOI:** 10.1002/mrm.27851

**Published:** 2019-07-18

**Authors:** Nadège Corbin, Julio Acosta‐Cabronero, Shaihan J. Malik, Martina F. Callaghan

**Affiliations:** ^1^ Wellcome Centre for Human Neuroimaging UCL Queen Square Institute of Neurology, University College London London United Kingdom; ^2^ School of Biomedical Engineering & Imaging Sciences King's College London London United Kingdom

**Keywords:** $B_{1}^{+}$ mapping, Bloch‐Siegert shift, GLM, interleaved acquisition, multi‐echo readout, RF spoiling

## Abstract

**Purpose:**

Quantitative MRI applications, such as mapping the T_1_ time of tissue, puts high demands on the accuracy and precision of transmit field ($B_{1}^{+}$) estimation. A candidate approach to satisfy these requirements exploits the difference in phase induced by the Bloch‐Siegert frequency shift (BSS) of 2 acquisitions with opposite off‐resonance frequency radiofrequency pulses. Interleaving these radiofrequency pulses ensures robustness to motion and scanner drifts; however, here we demonstrate that doing so also introduces a bias in the $B_{1}^{+}$ estimates.

**Theory and Methods:**

It is shown here by means of simulation and experiments that the amplitude of the error depends on MR pulse sequence parameters, such as repetition time and radiofrequency spoiling increment, but more problematically, on the intrinsic properties, T_1_ and T_2_, of the investigated tissue. To solve these problems, a new approach to BSS‐based $B_{1}^{+}$ estimation that uses a multi‐echo acquisition and a general linear model to estimate the correct BSS‐induced phase is presented.

**Results:**

In line with simulations, phantom and in vivo experiments confirmed that the general linear model‐based method removed the dependency on tissue properties and pulse sequence settings.

**Conclusion:**

The general linear model‐based method is recommended as a more accurate approach to BSS‐based $B_{1}^{+}$ mapping.

## 1 INTRODUCTION

Knowledge of the spatial distribution of the radiofrequency (RF) transmit field ($B_{1}^{+}$) is crucial to many MRI applications. Moderate accuracy may suffice when setting transmitter gain^1^ or calibrating multi‐channel systems.^2^ However, very high accuracy and precision are required for many quantitative MRI applications, e.g., mapping the longitudinal relaxation rate to characterize cortical myelination.^3^, ^4^

Phase‐based methods may be preferred as they are theoretically insensitive to T_1_ relaxation effects which often bias magnitude‐based methods, especially at short repetition time (TR). In the Bloch‐Siegert (BS)^5^, ^6^ approach, an off‐resonance RF pulse leads to the Bloch‐Siegert frequency shift (BSS), and an associated phase accumulation, which is proportional to the square of the pulse amplitude thereby encoding the $B_{1}^{+}$ field. This technique performed favorably in a recent review of the accuracy, precision and practicality of a range of prominent $B_{1}^{+}$ mapping techniques,^7^ and has been shown to be less sensitive to *B*_0_ inhomogeneities and chemical shifts^8^ than other phase‐based methods.

The BSS technique is flexible and can be integrated into a multitude of pulse sequences, such as 2D^9^ or 3D gradient echo (GRE),^8^, ^10^, ^11^ interleaved echo planar imaging, spiral GRE,^12^ and spin echo^13^, ^14^, ^15^, ^16^ acquisitions. The BSS technique, as typically implemented, requires two acquisitions with opposite off‐resonance frequencies. By subtracting the 2 phase images, the BSS effect is enhanced and unrelated phase components are removed, e.g., phase accumulated across echo time (TE) due to B_0_ inhomogeneity or chemical shifts, due to eddy currents, or any initial phase due to the transmitting and receiving coils. This subtraction also has the advantage of removing the effect of B_0_ inhomogeneity on the BSS, up to first order.^9^ These 2 acquisitions can either be played out sequentially or by interleaving the opposite off‐resonance frequencies. Previous reports have shown the interleaved approach to be more robust to motion^17^ and magnetic field drift.^10^

In this work, we focus on a 3D spoiled GRE implementation and demonstrate, through both simulations and experiments, that alternating the sign of the off‐resonance frequency from shot to shot in an interleaved manner disturbs the steady‐state and introduces an additional phase difference between the 2 acquisitions, especially at short TR. We show that this additional phase difference leads to bias in the $B_{1}^{+}$ map that depends on the relaxation parameters of the studied tissue, the specifics of the RF spoiling regime and the actual $B_{1}^{+}$ amplitude. We additionally propose and validate a modified BSS‐based approach that removes these dependencies. The solution consists of a multi‐echo acquisition in which several echoes are acquired before and after the BS pulse and modeling the phase evolution with a general linear model (GLM). We demonstrate that this GLM‐based approach to isolating the BSS phase allows the interleaved approach to be used without introducing any error, extending the acquisition time, increasing the specific absorption rate (SAR) or reducing the sensitivity.

## 2 THEORY

In line with Sacolick et al,^9^ and as detailed in the Supporting Information, which is available online, the BS phase introduced by an RF pulse with peak amplitude $B_{1}^{p}$ and normalized shape $B_{1}^{\mathrm{norm}}$, is proportional to the square of the peak pulse amplitude:

(1) $$\Phi_{\mathrm{BSS}}={\left(B_{1}^{p}\right)}^{2}\int_{0}^{T}{\left(\gamma B_{1}^{\mathrm{norm}}\left(t\right)\right)}^{2}*\left(\left[\frac{1}{2\omega_{\mathrm{off}}}-\frac{\Delta \omega_{B_{0}}}{2\omega_{\mathrm{off}}^{2}}\right]+O\left(\Delta \omega_{B_{0}}^{2}\right)\right)dt$$

$\omega_{\mathrm{off}}$ is the off‐resonance frequency of the pulse and $\Delta \omega_{B_{0}}$ is the local field inhomogeneity, both in Hz.

### 2.1 The classic method: Isolating the BSS phase by subtraction

The classic approach to BSS‐based $B_{1}^{+}$ mapping consists of acquiring 2 datasets with BS pulses of opposite off‐resonance frequency (i.e., $+\omega_{\mathrm{off}}$ and $-\omega_{\mathrm{off}}$). The phase difference between these is:

(2) $$\Phi_{BSS_{\mathrm{Diff}}}={\left(B_{1}^{p}\right)}^{2}\int_{0}^{T}{\left(\gamma B_{1}^{\mathrm{norm}}\left(t\right)\right)}^{2}*\left(\left[\frac{1}{2\omega_{\mathrm{off}}}-\frac{\Delta \omega_{B_{0}}}{2\omega_{\mathrm{off}}^{2}}\right]-\left[-\frac{1}{2\omega_{\mathrm{off}}}-\frac{\Delta \omega_{B_{0}}}{2\omega_{\mathrm{off}}^{2}}\right]+O\left(\Delta \omega_{B_{0}}^{2}\right)\right)dt$$

Because the first order terms that depend on $\Delta \omega_{B_{0}}$ cancel, this expression simplifies to:

(3) $$\Phi_{BSS_{\mathrm{Diff}}}={\left(B_{1}^{p}\right)}^{2}\int_{0}^{T}\frac{{\left(\gamma B_{1}^{\mathrm{norm}}\left(t\right)\right)}^{2}}{\omega_{\mathrm{off}}}dt+O\left(\Delta \omega_{B_{0}}^{2}\right)$$

Previously, this subtraction was assumed to also remove any phase accumulated from other sources, such as eddy‐currents, transmit/receive‐related phase offsets, chemical shifts, and local B_0_ inhomogeneities. However, crucially, this is only true if the additional phase components are identical for each of the off‐resonance frequencies. If this assumption is violated, the $B_{1}^{+}$ estimate will be erroneous.

### 2.2 The GLM method: Isolating the BSS phase by modeling a multi‐echo acquisition

We propose an alternative to the classic BSS approach that computes accurate $B_{1}^{+}$ maps even if conditions vary between the two off‐resonance frequency acquisitions. This approach relies on two novel features: a dual‐offset multi‐echo sequence and a GLM.

#### 2.2.1 Dual‐offset multi‐echo sequence

**Figure 1:**
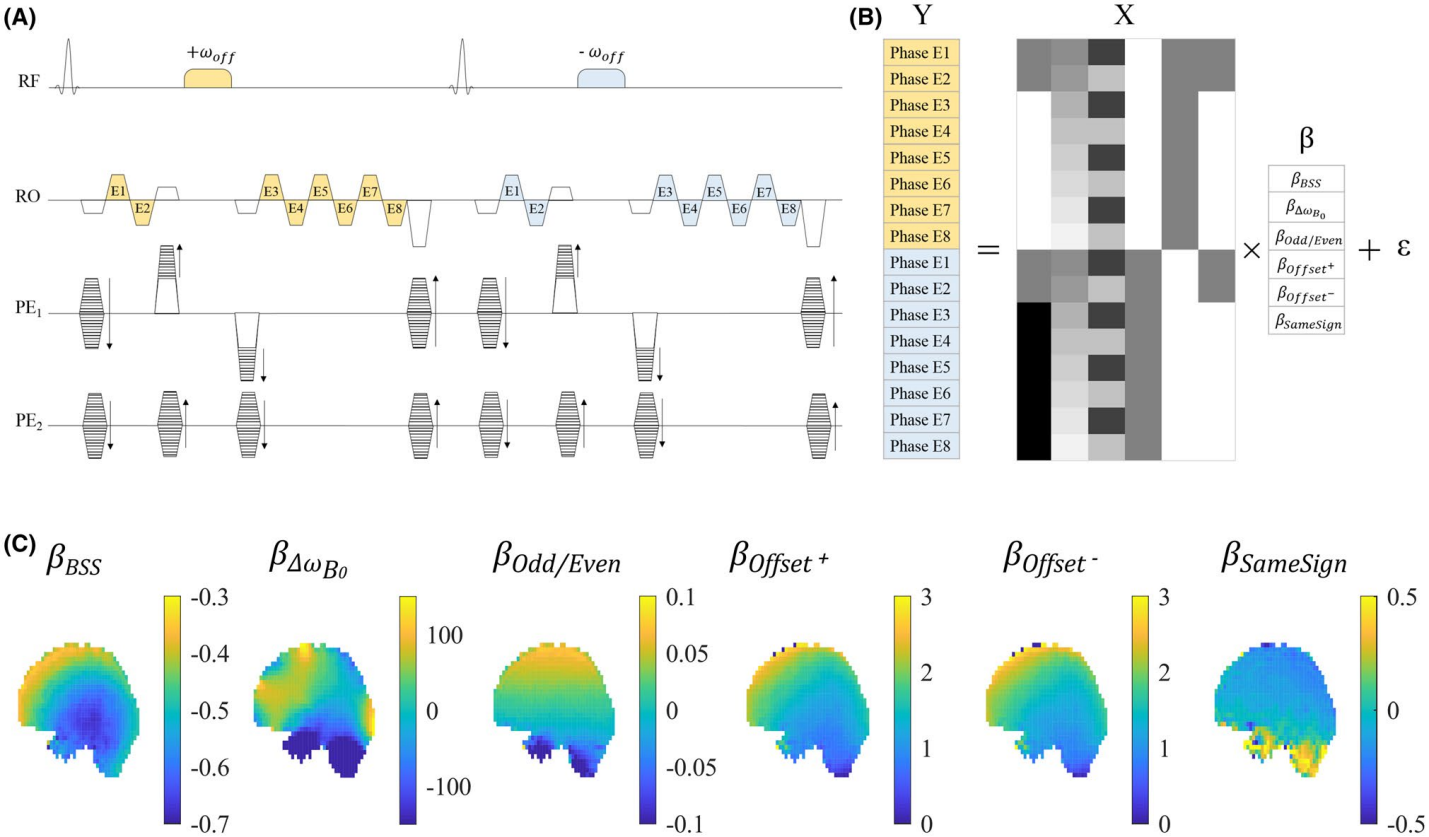
(A) The sequence diagram of the modified 3D multi‐echo GRE for BSS‐based $B_{1}^{+}$ mapping. Two echoes are acquired before, and 6 echoes after, the BS pulse, which is flanked by crushers in 1 phase‐encoding direction ($PE_{1})$ to destroy any inadvertent on‐resonance excitation and minimize dependence on excitation flip angle. The gradients on each axis are balanced before the BS pulse. In this example, 8 echoes are acquired for each off‐resonance frequency, resulting in a total of 16 phase images from which the $B_{1}^{+}$ efficiency is mapped. (B) A GLM is used to model the phase variation across TEs. (C) Typical maps of model coefficients obtained in vivo exemplify the phase accrued due to the BSS ($\beta_{\mathrm{BSS}})$, B_0_ field inhomogeneity, $\left(\beta_{\Delta \omega_{B_{0}}}\right)$, alternating readout (RO) polarity, $\left(\beta_{Odd/Even}\right)$, initial phase offsets specific to the off‐resonance frequency of the pulse, $\left(\beta_{Offset^{+}},\beta_{Offset^{-}}\right)$, and any additional phase due to the presence of the block of crushers and the BS pulse that is independent of the sign of the off‐resonance frequency of the pulse, $\left(\beta_{\mathrm{SameSign}}\right)$

In the modified BSS‐based $B_{1}^{+}$ mapping sequence (Figure 1), multiple echoes are acquired after one excitation pulse. Two echoes, after the BS pulse, have previously been used^18^ to concurrently compute the B_0_ inhomogeneity, whereas here multiple echoes are acquired before and after the BS pulse. As in the classic method, a second acquisition is performed with the opposite off‐resonance frequency, either sequentially or interleaved.

#### 2.2.2 GLM

The GLM approach models the phase data, Y, as multiple linear sources of phase accumulation over time: Y=Xβ+ϵ.

Each row of the model matrix X, corresponds to a single echo. The following explanatory variables model distinct sources of phase evolution in separate columns:

- $X_{\mathrm{BSS}}$: Models the phase accumulated due to the BS pulse, specifically the first term of the sum in Equation 1 (only). This phase should only be present after the BS pulse, and should change sign with the off‐resonance frequency. The regressor consists of zeros for echoes before the BS pulse and either 1 or −1 afterward depending on the BS pulse frequency.
- $X_{\Delta \omega_{B_{0}}}$: Models the phase accumulated due to local $B_{0}$ inhomogeneities. Regressor values are TEs.
- $X_{\mathrm{Odd}/\mathrm{Even}}$: Models the phase difference between odd and even echoes due to eddy currents generated by the bipolar readout gradients. $X_{Odd/Even}$ is 1 for odd echoes and ‐1 for even echoes.
- $X_{{\mathrm{Offset}}^{+}}\;\;\text{and}\;X_{{\mathrm{Offset}}^{-}}$: Models the initial phase offset of the acquisitions with positive and negative off‐resonance frequencies, respectively. $X_{Offset^{+}}$ is 1 for all echoes from a TR with a BS pulse with positive off‐resonance frequency, and 0 for all echoes from a TR with a BS pulse with negative off‐resonance frequency; vice versa for $X_{Offset^{-}}$.
- $X_{\mathrm{SameSign}}$: Models phase consistently accumulated during the BS pulse and the crushers, regardless of the sign of the BS pulse's off‐resonance frequency. This includes the second term in Equation 1 and, for example, any phase due to eddy currents generated by the crushers. $X_{\mathrm{SameSign}}$ is 0 for echoes before the BS pulse and 1 for echoes after the BS pulse, regardless of the pulse's off‐resonance frequency.

The parameters β constitute the regression coefficients and are estimated voxel‐wise by means of the weighted least‐square approach: $\hat{\beta }={\left(X^{T}WX\right)}^{-1}X^{T}WY$. W is a diagonal weighting matrix in which the weights are the magnitude of the echoes. ${\hat{\beta }}_{\mathrm{BSS}}$, the parameter of interest, is considered an estimate of $\Phi_{\mathrm{BSS}}$. *ε* is an error term.

## 3 METHODS

### 3.1 Numerical simulations

Simulations were used to evaluate the difference, both before and after the BS pulse, between two acquisitions with opposite off‐resonance frequencies. Any difference present at this point would introduce an error in $B_{1}^{+}$ estimated with the classic method. A typical GRE acquisition was simulated by a series of matrix operations (described in Supporting Information). Several configurations were investigated.

#### 3.1.1 Effect of RF Spoiling increment

RF spoiling modifies the phase of the excitation pulse and the BS pulse (ϕ) so that the phase difference between successive TR periods increases linearly by a constant amount $\phi_{\mathrm{BaseInc}}$.

The impact of the RF spoiling increment was investigated by changing $\phi_{\mathrm{BaseInc}}$ from 0° to 180° in 10° steps. A phase increment of 0 corresponds to no RF spoiling (i.e., ϕ=0 for all pulses).

#### 3.1.2 Sequential or interleaved

For an interleaved acquisition, the sign of $\omega_{\mathrm{off}}$ was alternated between successive BS pulses. For sequential acquisitions, the sign was switched after half the total number of pulses $\left(\frac{N_{\mathrm{exc}}}{2}\right)$, at which point RF spoiling was also reset.

#### 3.1.3 Effect of *TR, T_1_, T_2_,* and $B_{1}^{p}$

Extreme values were used to test the impact of sequence parameters, $B_{1}^{p}\in \left[8,11\right]\mu T$, $TR\in \left[35,100\right]ms$, relaxation parameters, $T_{1}\in \left[550,1350\right]ms$, and $T_{2}\in \left[70,100\right]ms$ on the $B_{1}^{+}$ estimate.

The estimated $B_{1}^{+}$ amplitude was calculated as follows having isolated the first term (only) of Equation 1:

(4) $$B_{1}^{p}=\sqrt{\frac{\Phi_{\mathrm{BSS}}}{\int_{0}^{T}\frac{{\left(\gamma B_{1}^{\mathrm{norm}}\left(t\right)\right)}^{2}}{2\omega_{\mathrm{off}}}dt}}$$

For the Classic method, $\Phi_{\mathrm{BSS}}$ was taken to be half the difference in phase accumulated after the BS pulse of the acquisitions with opposite off‐resonance frequency, and termed $\Phi_{\mathrm{BSS}}^{\mathrm{Classic}}$ (see Supporting Information).

For the GLM method, $\Phi_{\mathrm{BSS}}$ was taken to be the mean absolute phase accrued during the BS pulses of the acquisitions with opposite off‐resonance frequency, and termed $\Phi_{\mathrm{BSS}}^{\mathrm{GLM}}$ (see Supporting Information).

### 3.2 MRI measurements

#### 3.2.1 MR pulse sequence

Measurements were performed at 3T (Siemens Prisma) using a body coil for transmission and a 32‐channel head coil for signal reception using an in‐house MR pulse sequence (Figure 1). A Fermi pulse of duration T = 2 ms imparted the BSS after the second echo. Acquiring 2 echoes before the BS pulse served to minimize the correlation of the $X_{\mathrm{BSS}}$ regressor with other regressors while maintaining a reasonable TE for the echo after the BS pulse. The encoding gradients on all axes were balanced immediately before the BS pulse, to ensure the same dephasing state for the magnetization across TRs, and played again just after. Crusher gradients were played out either side of the BS pulse, concurrently with the balancing/phase‐encoding gradients (on PE_1_), to crush any undesired on‐resonance excitation and to minimize any dependence on the excitation flip angle. As demonstrated by Duan et al,^18^ perfect dephasing of the transverse magnetization before the BS pulse is required to fully remove any such dependence. Sensitivity to nonideal conditions is reduced by using a high crusher moment because the greater the dephasing, the smaller the dependence on the excitation flip angle. Therefore, a relatively large crusher moment,^19^ designed to generate a theoretical dephasing of 6π rad across a voxel, was used. An excitation flip angle of 15°, corresponding to the Ernst angle for a TR of 35 ms and a T_1_ of 1000 ms, was chosen to maximize the precision of the $B_{1}^{+}$ mapping.^18^ Although this may be somewhat sub‐optimal from a precision perspective for the phantom acquisitions and the long TR in vivo acquisitions, it was used for all acquisitions to ensure consistency.

For sequential acquisitions, the positive and negative off‐resonance frequency pulses were played out in consecutive blocks. In the interleaved case, the off‐resonance frequency was alternated across successive TR periods. To achieve steady state, 200 dummy cycles were executed before each block in the sequential case, 400 cycles were used at the outset of the interleaved case (to match acquisition times), and in both cases a spoiler gradient, set to reach a dephasing of 6π at the end of the TR, was applied in the readout direction after the last echo. The RF spoiling was reset at the end of the first block in the sequential case.

Table 1 lists sequence parameters of all experiments.

**Table 1:** List of the parameters of the phantom and in vivo acquisitions

TR	Repetition time	35 ms; 100 ms for the reference
TE	Echo times	[2.38 4.68 11.42 13.72 16.02 18.32 20.62 22.92] ms
$\omega_{\mathrm{off}}$	Off‐resonance frequency of the BS pulse	2 kHz
$\alpha_{\mathrm{BS}}$	Flip angle of the BS pulse	260 °
T	Duration of the BS pulse	2 ms
α	Excitation flip angle	15 °
$\phi_{\mathrm{BaseInc}}$	Increment of the RF spoiling	[0° 10° 20° 50° 60° 70° 90° 110° 120° 130° 160° 170° 180°] for Phantom experiment 1
		[0° 90° 120°] for Phantom experiment 2 and in vivo experiment
$\Omega_{1}^{\max}$	Crusher gradient dephasing moment	6π rad
$\Omega_{2}^{\max}$	Spoiling moment per TR	6π rad
	Field of view	256 × 224 × 192 mm^2^
	Acquisition matrix	64 × 56 × 48
	Voxel size	4 × 4 × 4 mm^3^

#### 3.2.2 $B_{1}^{+}$ map estimation

All data, including $B_{1}^{+}$ maps, were reconstructed in real time using in‐house code implemented in Gadgetron.^20^ An apodizing filter was applied to the raw k‐space data along each dimension to minimize ringing artefacts. After Fourier transformation the images were adaptively combined across coil elements.^21^

Two $B_{1}^{+}$ maps were computed for each dataset using the Classic and GLM methods, respectively. For the Classic method, the phase of the third echoes, which were acquired after the positive and negative off‐resonance frequency BS pulses, were subtracted to estimate the BSS phase: $\Phi_{\mathrm{BSS}}=\Phi_{\mathrm{Diff}}/2$. For the GLM method, the phase images for each off‐resonance frequency were temporally unwrapped, by spatially unwrapping the differences between successive echo pairs,^22^ and cumulatively adding these to the phase of the first echo. The phase images were subsequently used to estimate the parameters of the GLM (Figure 1). The BSS phase $\Phi_{\mathrm{BSS}}$ was captured by the first regressor $X_{\mathrm{BSS}}$ of the model matrix such that $\Phi_{\mathrm{BSS}}={\hat{\beta }}_{\mathrm{BSS}}$.

For both methods, $B_{1}^{p}$ was computed on a voxel‐wise basis using Equation 4.

#### 3.2.3 Phantom experiments

$B_{1}^{+}$ maps were acquired on an FBIRN gel phantom.^23^ Experiments 1 and 2 used both the Classic and GLM processing methods to construct $B_{1}^{+}$ maps. $B_{1}^{+}$ errors were quantified as the percent difference of the estimated $B_{1}^{+}$ with respect to a reference $B_{1}^{+}$ map..

##### Phantom experiment 1: Comparing processing approaches

This experiment probed the impact of the processing method as a function of the RF spoiling increment, $\phi_{\mathrm{BaseInc}}\in \left[0;\;10;\;20;\;50;\;60;\;70;\;90;\;110;\;120;\;130;\;160;\;170;\;180\right])$. The interleaved acquisition scheme was used for all scans. $B_{1}^{+}$ maps were reconstructed with the Classic and GLM methods. The voxel‐wise difference between the two $B_{1}^{+}$ maps (relative to the GLM method) was computed, and summarized by the mean and standard deviation across the phantom. A TR of 35 ms was used.

##### Phantom experiment 2: Comparing sequential and interleaved approaches

A reference $B_{1}^{+}$ map was obtained, by means of the Classic method, using a sequential acquisition with $\phi_{\mathrm{BaseInc}}=90^{\circ }$. Four interleaved acquisitions were performed with: (1) no RF spoiling, TR = 35 ms; (2) $\phi_{\mathrm{BaseInc}}=120^{\circ }$, TR = 35 ms; (3) $\phi_{\mathrm{BaseInc}}=90^{\circ }$ , TR = 35 ms; and (4) $\phi_{\mathrm{BaseInc}}=120^{\circ }$, TR = 100 ms.

$B_{1}^{+}$ maps were created using both Classic and GLM methods and compared with the reference map. Histograms of the relative difference in $B_{1}^{+}$ estimates were calculated for each RF spoiling condition and processing method.

#### 3.2.4 In vivo experiments

Three healthy participants (2 males, 28‐40 years) were scanned. Five datasets were acquired per participant, with TR = 35 ms unless otherwise stated: (1) Interleaved, with $\phi_{\mathrm{BaseInc}}=120^{\circ }$ and TR = 100 ms. This produced the reference $B_{1}^{+}$ map. (2) Interleaved without RF spoiling. (3) Interleaved with $\phi_{\mathrm{BaseInc}}=120^{\circ }$. (4) Interleaved with $\phi_{\mathrm{BaseInc}}=90^{\circ }$. And (5) Sequentially with $\phi_{\mathrm{BaseInc}}=120^{\circ }$.

Two $B_{1}^{+}$ maps were estimated for each scan using the Classic and GLM methods, respectively. The percent difference in $B_{1}^{+}$ was calculated with respect to the reference map for the same processing method. To compare processing methods, the percent difference between the two $B_{1}^{+}$ maps derived from the reference acquisition was also computed, with respect to that obtained with the Classic method.

## 4 RESULTS

### 4.1 Numerical simulations

#### 4.1.1 Effect of acquisition order and RF spoiling

**Figure 2:**
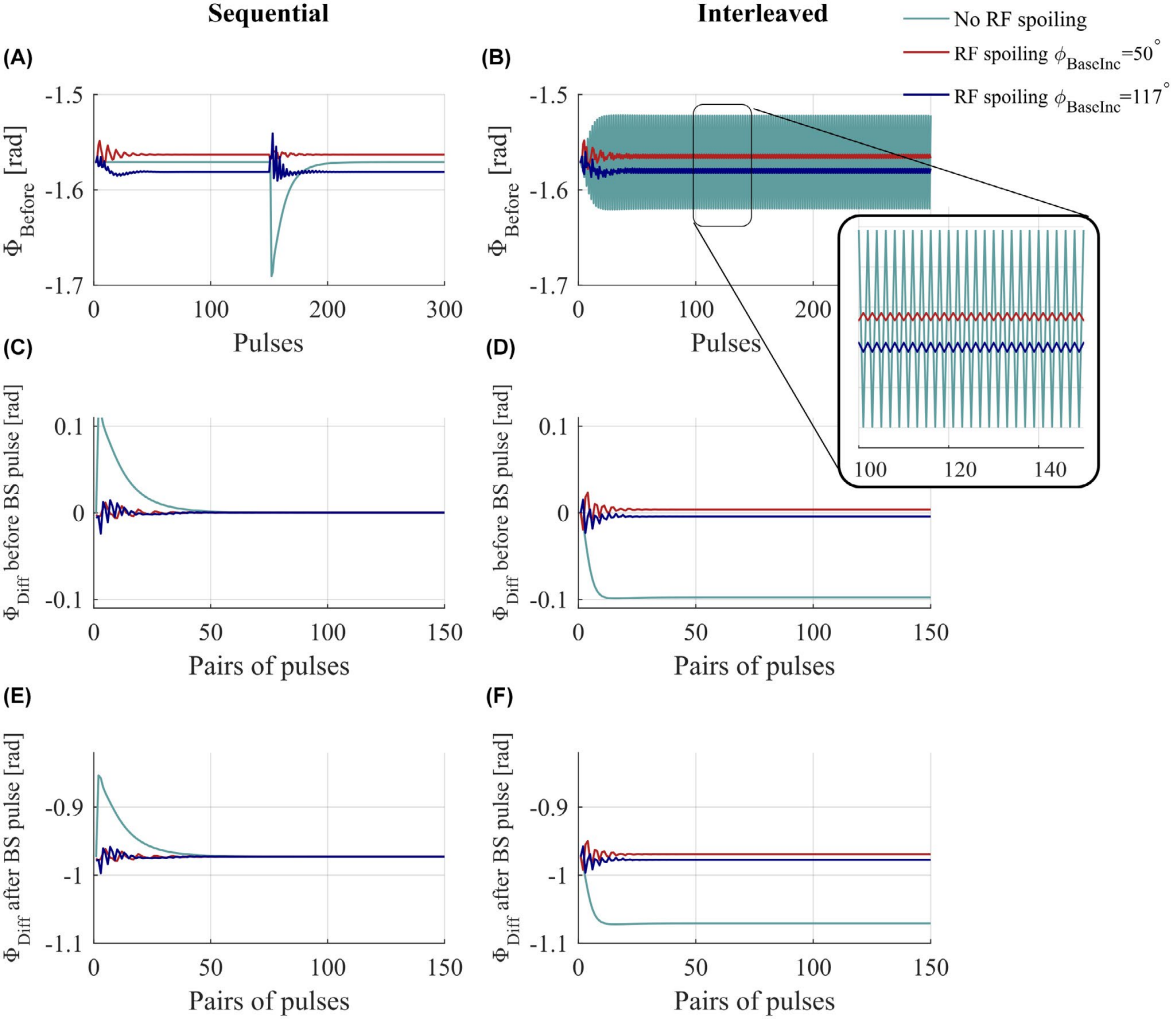
Numerical simulation results. Phase accrued before the BS pulse in case of sequential acquisitions (A) or interleaved acquisitions (B), in the presence (red and blue curve) of RF spoiling $\left(\Phi_{\mathrm{BaseInc}}=50^{\circ }\;\text{and}\;\;117^{\circ }\right)$ or without RF spoiling (green). Phase difference before the pulses of the two acquisitions with opposite frequencies (C,D) and phase difference after the BS pulse (E,F)

For *sequential* acquisition ordering, once steady‐state was reached for each off‐resonance frequency block (Figure 2A), the phase difference between these was zero before the BS pulse (Figure 2C). It was nonzero after the BS pulse (Figure 2E) because it contained the BSS phase. However, this BSS phase estimate remained constant over time and was independent of the RF spoiling condition.

For *interleaved* acquisitions, the phase before the BS pulse varied from pulse to pulse regardless of pulse number (Figure 2B). This temporal variance caused a nonzero phase difference between the interleaved acquisitions with opposite BS pulse frequencies, both before (Figure 2D) and after (Figure 2F) the BS pulse. Furthermore, the phase difference after the BS pulse, i.e., the estimate of the BSS phase, differed from the phase difference obtained with the sequential approach and depended strongly on the RF spoiling conditions.

#### 4.1.2 Effect of RF spoiling increment

**Figure 3:**
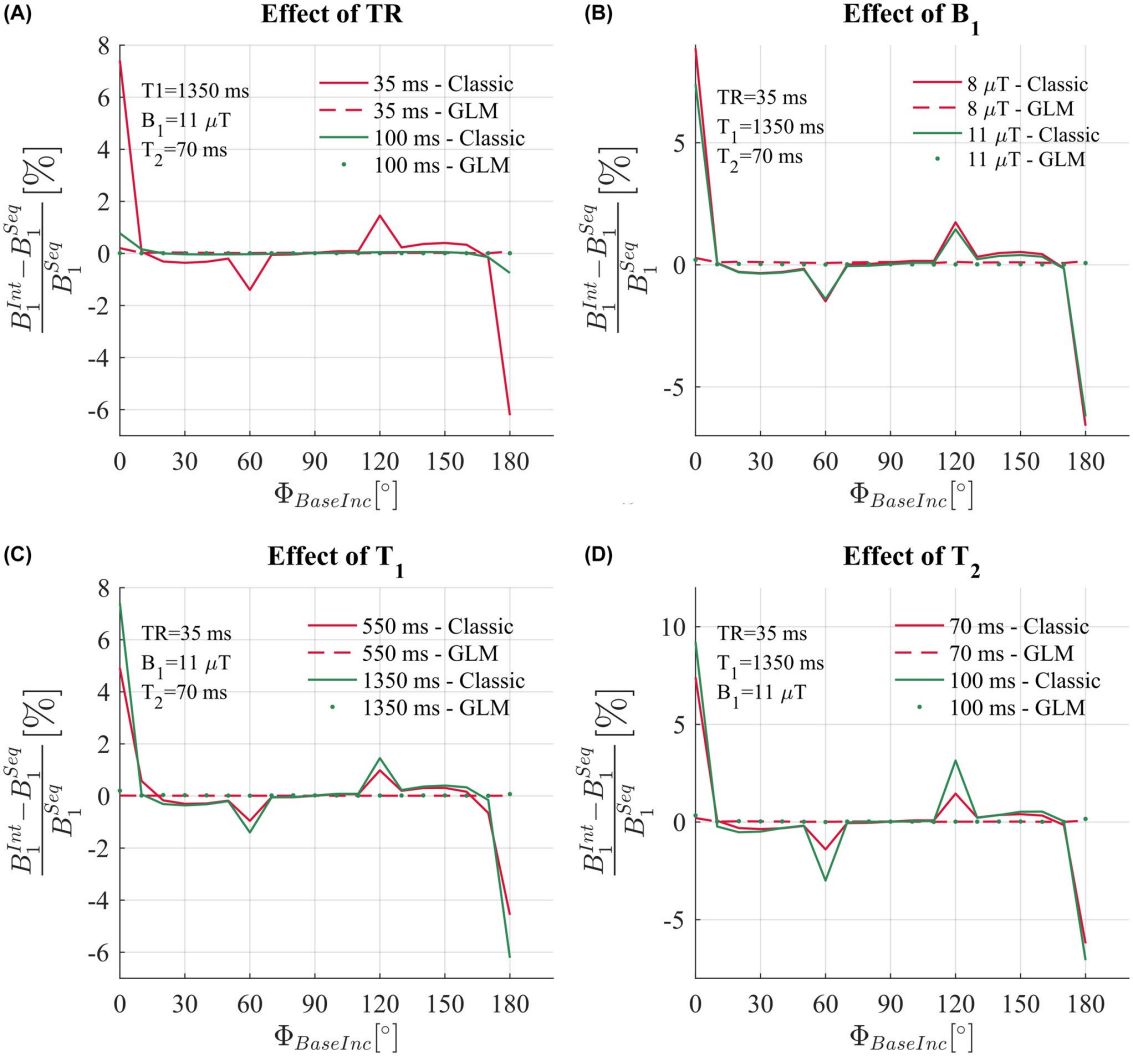
Numerical simulation results. Relative error of the estimate of $B_{1}^{+}$ obtained with interleaved acquisitions $B_{1}^{\mathrm{Int}}$ with respect to the estimated $B_{1}^{+}$ obtained with sequential acquisitions $B_{1}^{\mathrm{Seq}}$. $B_{1}^{\mathrm{Int}}$ is computed in 2 ways, the Classic method solid lines, and the GLM method (Equation 4), dashed and dotted lines. $B_{1}^{\mathrm{Seq}}$ is only computed with the Classic method. The error is observed over a large range of RF spoiling increment $\left(\Phi_{\mathrm{BaseInc}}\in \left[0:10:180\right]\right)$, for 2 TR values (A), 2 $B_{1}^{+}$ values (B), 2 T_1_ values (C), and 2 T_2_ values (D)

The error in the BSS phase resulting from simulating an interleaved acquisition and using the Classic method depended strongly on the RF spoiling increment used (Figure 3). The greatest errors were predicted for phase increments of 0° (equivalent to no RF spoiling) and 180°. Large errors were also predicted for phase increments of 60° and 120°, whereas no error was predicted at 90°.

#### 4.1.3 Effect of TR, $B_{1}^{+}$, T_2_, and T_1_

Increasing TR greatly reduced the predicted error in $\Phi_{\mathrm{BSS}}$ (Figure 3A). Longer T_1_ times led to larger predicted errors, but had less impact than TR (Figure 3C). Similarly, longer T_2_ times predicted larger errors (Figure 3D), especially with phase increments of 60 and 120° where the predicted error was already large.

The amplitude of the BS pulse also had a small impact whereby the *relative* error was predicted to be lower for higher amplitude pulses (Figure 3B). Note that in this case $B_{1}^{+}$ also increases, so while the predicted relative error decreased, the absolute error is actually increased.

#### 4.1.4 Impact of GLM method for BSS estimation

The numerical simulations predicted that these errors were removed by using the GLM approach. As a result, the derived $B_{1}^{+}$ estimates were predicted to be stable and agree with the $B_{1}^{+}$ estimated from the sequential case using the Classic method. This was the case regardless of the RF spoiling conditions, TR, T_1_, T_2_, or BS pulse amplitude.

### 4.2 Phantom experiments

*Phantom experiment 1: Comparing processing approaches*

**Figure 4:**
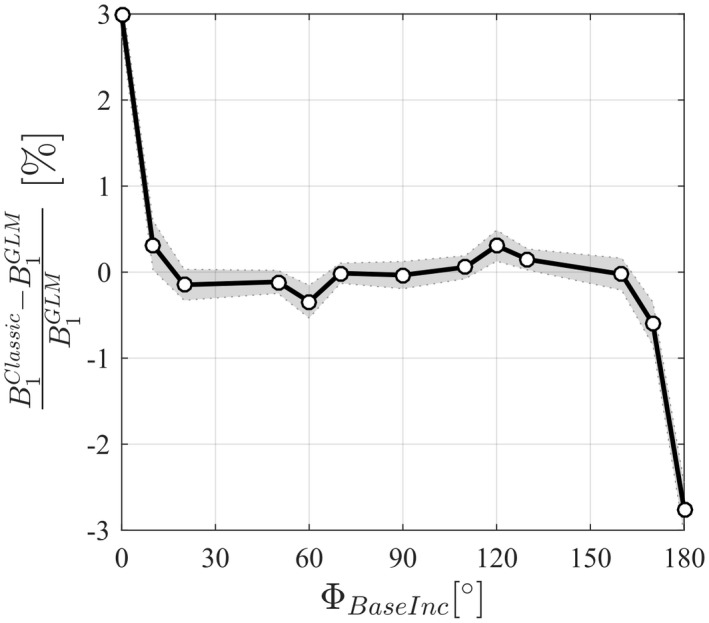
Relative difference of the $B_{1}^{+}$ map of a phantom obtained with the Classic method $B_{1}^{\mathrm{Classic}}$, with respect to the $B_{1}^{+}$ map reconstructed with the GLM method. Each circle is the average difference across the phantom. $B_{1}^{+}$ maps were obtained with interleaved acquisitions and repeated over a range of RF spoiling increment $\Phi_{\mathrm{BaseInc}}={\left[0\;10\;20\;50\;60\;70\;90\;110\;120\;130\;160\;170\;180\right]}^{\circ }$

With RF spoiling increments of 0 and 180°, the $B_{1}^{+}$ estimates were very different depending on the processing method used (Figure 4), but these estimates agreed when the RF spoiling increment was 90°. Two further peaks in the discrepancy were observed with RF spoiling increments of 60° and 120°. The difference observed without RF spoiling ($\Phi_{\mathrm{BaseInc}}=0^{\circ })$ was 3.0%.

*Phantom experiment 2: Comparing sequential and interleaved approaches*

**Figure 5:**
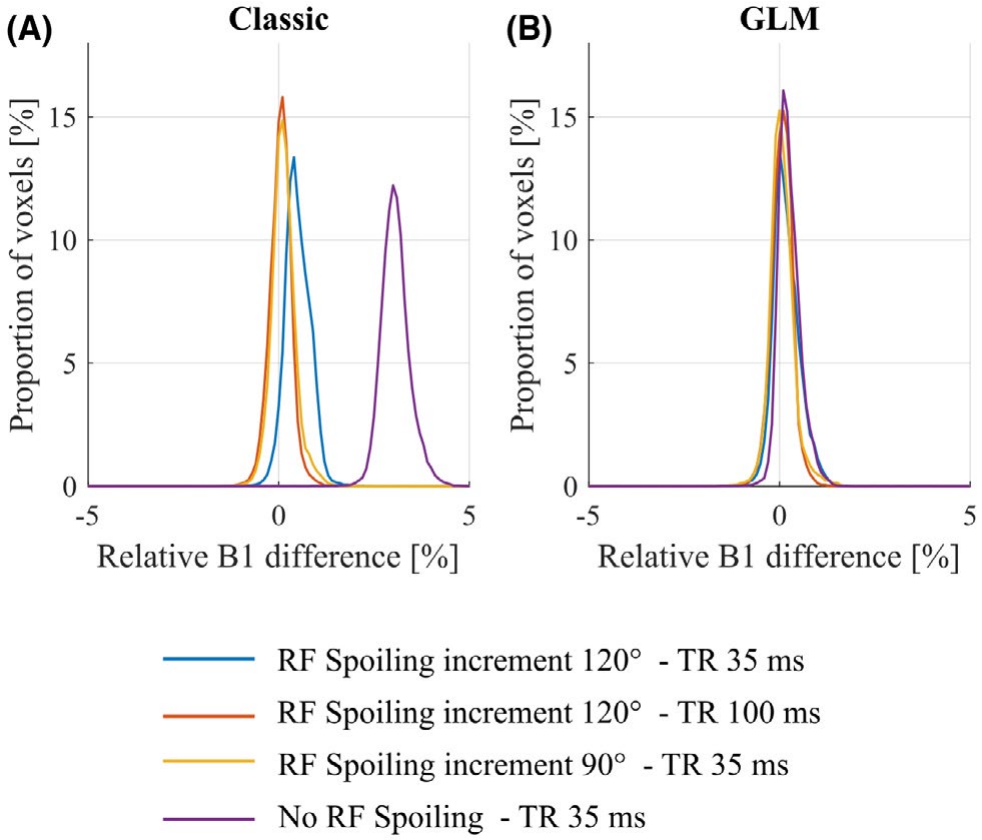
Histograms of the difference in $B_{1}^{+}$ measured in a phantom with an interleaved acquisition scheme relative to the reference $B_{1}^{+}$ map acquired with a sequential acquisition and processed using the Classic method. The $B_{1}^{+}$ maps were calculated using either the Classic (A) or GLM (B) method. The data were acquired with a short TR (35 ms) without (*purple*) or with RF spoiling ($\Phi_{\mathrm{BaseInc}}=120^{\circ }\left(\mathrm{blue}\right)\;and\;\Phi_{\mathrm{BaseInc}}=90^{\circ }\left(\mathrm{yellow}\right)$), or a long TR (100 ms) and RF spoiling ($\Phi_{\mathrm{BaseInc}}=120^{\circ })\left(\mathrm{red}\right)$. The reference $B_{1}^{+}$ map, with respect to which the error was calculated, was acquired with sequential ordering and RF spoiling ($\Phi_{\mathrm{BaseInc}}=90^{\circ })$ and processed using the Classic method

$B_{1}^{+}$ maps estimated using the Classic method from interleaved data and sequential data (the reference case) did not agree. The largest bias was observed with no RF spoiling (median (interquartile range) of 3.04% (0.45%), Figure 5A). The bias was greatly reduced by RF spoiling with $\phi_{\mathrm{BaseInc}}=120^{\circ }$ (−0.47% (0.44%)). Negligible bias was observed with $\phi_{\mathrm{BaseInc}}=90^{\circ }$ (−0.11% (0.36%) or with a longer TR of 100 ms and $\phi_{\mathrm{BaseInc}}=120^{\circ }$ (0.06% (0.34%)).

The biases observed without RF spoiling and with $\phi_{\mathrm{BaseInc}}=120^{\circ }$ relative to the sequential case were removed when the GLM method was used to process the same interleaved data (Figure 5B; 0.21% (0.36%) for no RF spoiling and 0.13% (0.44%) for RF spoiling with $\phi_{\mathrm{BaseInc}}=120^{\circ }$).

### 4.3 In vivo experiments

The reference acquisition, which used interleaved ordering, a longer TR of 100 ms and an RF spoiling increment of $\phi_{\mathrm{BaseInc}}=120^{\circ },$ produced consistent $B_{1}^{+}$ maps with the Classic and GLM methods (see Figure 7C,F,I). The median (interquartile range) differences relative to the GLM method were: 0.09% (0.72%), 0.30% (0.97%) and 0.11% (0.60%) for participants 1, 2, and 3, respectively. However, higher $B_{1}^{+}$ values were observed in the ventricles of Participant 2 (see Figure 8) of the map computed with the classic method. Consistent with this observation, a tail in the histogram was also present for participant 2 (see Figure 7F).

Large bias was seen with respect to this reference when $B_{1}^{+}$ maps were estimated from interleaved data, acquired without RF spoiling, using the Classic method (see Figure 7A,D,G). Qualitatively, the bias was highly visible in the $B_{1}^{+}$ maps (see Figure 8A) following anatomical detail, and was greatest in the ventricles with long T_1_ and T_2_. In the difference map, strong bias was visible along the cortical ribbon (see Figure 8B). Median (interquartile range) differences were 4.52% (2.92%), 3.99% (2.48%), 3.86% (2.63%) for participants 1, 2, and 3, respectively. These biases were greatly reduced when the data were processed using the GLM method: −0.33% (1.78%), −0.10% (1.22%), and −0.35% (1.98%), respectively (see Figures 7 B,E,H and 8C,D).

The bias was greatly reduced when RF spoiling was used ($\Phi_{\mathrm{BaseInc}}\in \left[90^{\circ },120^{\circ }\right]$), and never exceeded 0.69%. However, systematically higher $B_{1}^{+}$ values were observed with $\Phi_{\mathrm{BaseInc}}=120^{\circ }$ compared with $\Phi_{\mathrm{BaseInc}}=90^{\circ }$ (see Figure 7, yellow and purple curves). This difference in $B_{1}^{+}$ values was greatly reduced when using the GLM method.

High variability in $B_{1}^{+}$ bias was observed when the sequential acquisition ordering was used (see Figure 7, blue curves) and artefacts were visible in the $B_{1}^{+}$ and difference maps (see Figure 8A‐D). This was the case regardless of the processing approach.

## 5 DISCUSSION

Efficient methods for mapping the $B_{1}^{+}$ transmit field with high accuracy and precision are prerequisite for demanding MRI applications, such as the quantification of the longitudinal relaxation rate.^3^ Biases in $B_{1}^{+}$ estimates may underlie intersite differences in relaxation rates,^24^ while uncertainty in the estimates will lower reproducibility.^4^ $B_{1}^{+}$ mapping based on the phase accrued due to the BSS has been reported to be an efficient technique for accurately estimating the spatial distribution of $B_{1}^{+}$ when compared with other magnitude or phase‐based techniques.^7^, ^25^

Our numerical simulations indicate that the sequential approach for acquiring the necessary BSS data will deliver a bias‐free estimate of the $B_{1}^{+}$ field. However, in agreement with previous reports,^10^, ^17^ our in vivo experiments show that this approach is sensitive to phase perturbations over time such as those caused by motion and scanner drifts. The resulting $B_{1}^{+}$ maps had visible artefacts and large biases (see Figure 8, right column). The greater robustness of the interleaved acquisition scheme has led to its adoption in more recent work using this technique.^11^, ^26^

In the interleaved case, however, our numerical simulations showed that the phase never reached steady‐state but rather a pseudo‐steady‐state that alternated between two conditions depending on the frequency of the preceding off‐resonance BS pulse. As a result, the difference in phase between interleaves is not solely due to the BSS effect and, therefore, does not match the phase difference of the sequential ordering scheme (Figure 2). The additional phase accrued biased the estimated BSS phase (Figure 3) and, therefore, the $B_{1}^{+}$ estimates in phantom and in vivo experiments. We have shown that the bias depends on intrinsic tissue properties (T_1_, T_2_) as well as sequence parameters (TR, RF spoiling increment, amplitude of the BS pulse).

Although the biases observed here are relatively small, their impact on the R_1_ estimate can be far greater. For example, the variable flip angle technique, widely used for whole brain R_1_ mapping^27^, ^28^, ^29^, ^30^ is highly sensitive to $B_{1}^{+}$ inhomogeneity and, therefore, requires correction. In this case, when estimating R_1_ from 2 weighted images with different flip angles, the accuracy of the $B_{1}^{+}$ estimate is crucial because it can be shown that a given bias in $B_{1}^{+}$ will lead to a bias in the R_1_ estimate that is at least twice as large and increases by even more for acquisitions with high flip angles or large error in the $B_{1}^{+}$ estimate. In fact, under certain conditions, such as in cerebrospinal fluid where the T_1_ and T_2_ are long, the $B_{1}^{+}$ bias can reach 8% (see Figure 8) when no RF spoiling is used, which would lead to a minimum of 16% bias in R_1_ with the variable flip angle technique. Hence, even small errors must be accounted for if accurate and robust R_1_ estimates are to be obtained.

Here, we have proposed and validated a novel acquisition and processing scheme for interleaved BSS‐based $B_{1}^{+}$ mapping that does not suffer from these biases. Crucially, multiple echoes are acquired either side of the BS pulse and a GLM framework is used to describe the phase evolution over time. The GLM models the effects of the BS pulse, B_0_ inhomogeneity, eddy currents and phase offsets both common to, and specific to, the positive and negative off‐resonance frequency interleaves. The bias observed with the Classic method results from the invalid assumption that the only difference between the 2 interleaves is the phase imparted by the BS pulse. Indeed, as demonstrated by the numerical simulations, a difference is already present before playing out the BS pulse (Figure 2D). The use of 2 distinct regressors ($X_{\mathrm{offset}}^{+}$ and $X_{\mathrm{offset}}^{-}$) in the model matrix of the GLM allows the 2 interleaves to differ, even before the BS pulse. This removes the bias in $B_{1}^{+}$ that would otherwise be present. Numerical simulations (Figure 3) and phantom experiments (Figure 4) confirm this, with both showing peak differences for RF spoiling increments of 0° (equivalent to no RF spoiling), 60°, 120°, and 180°.

**Figure 6:**
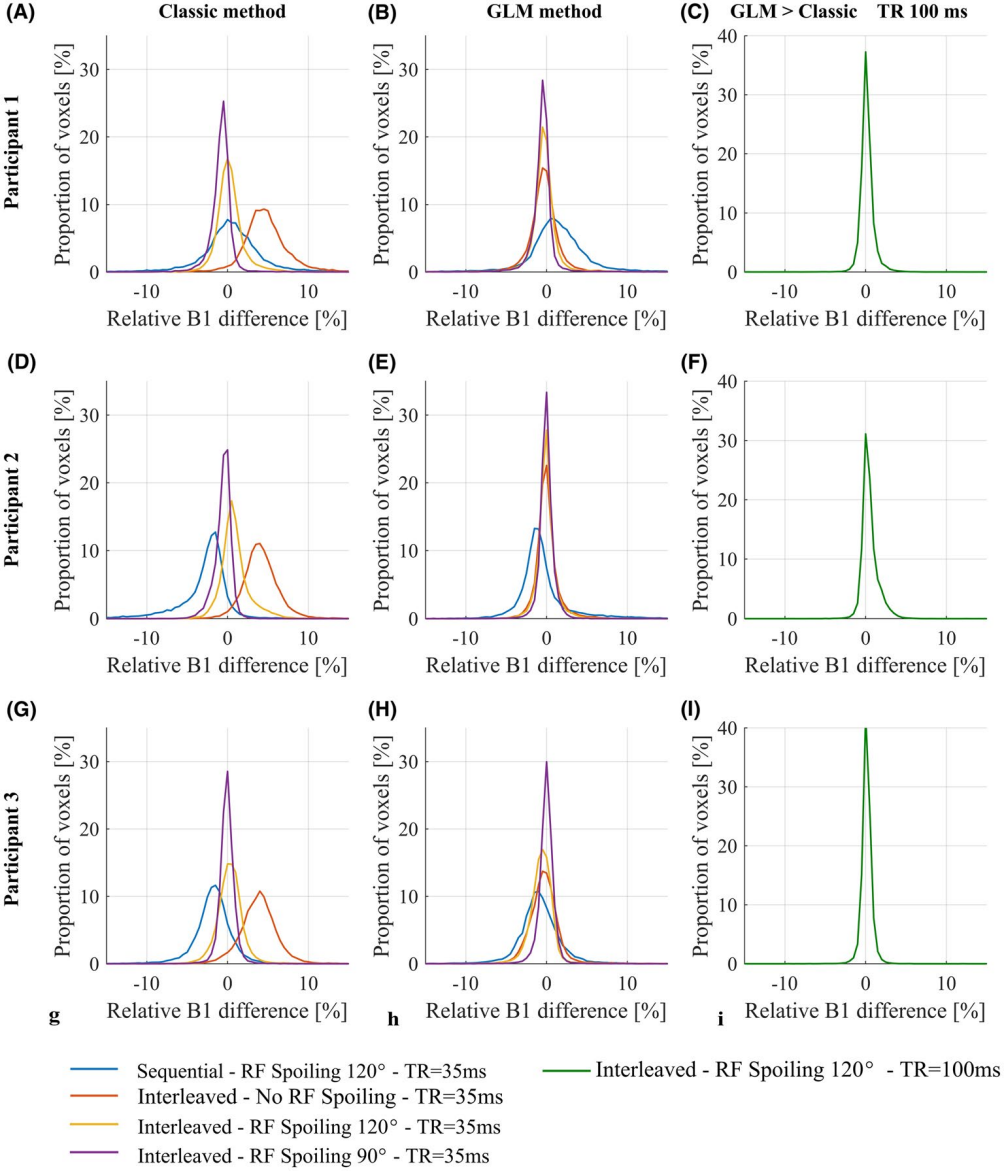
Histograms of the difference in $B_{1}^{+}$ relative to the reference $B_{1}^{+}$ map acquired in vivo with RF spoiling ($\Phi_{\mathrm{BaseInc}}=120^{\circ })$ with a TR of 100 ms, in interleaved order. The $B_{1}^{+}$ maps are either calculated with the classic method (A‐D‐G) or the GLM method (B‐E‐H). Percentage difference between the reference $B_{1}^{+}$ maps computed with the GLM and the Classic methods (C‐F‐I). Each row corresponds to a different participant

**Figure 7:**
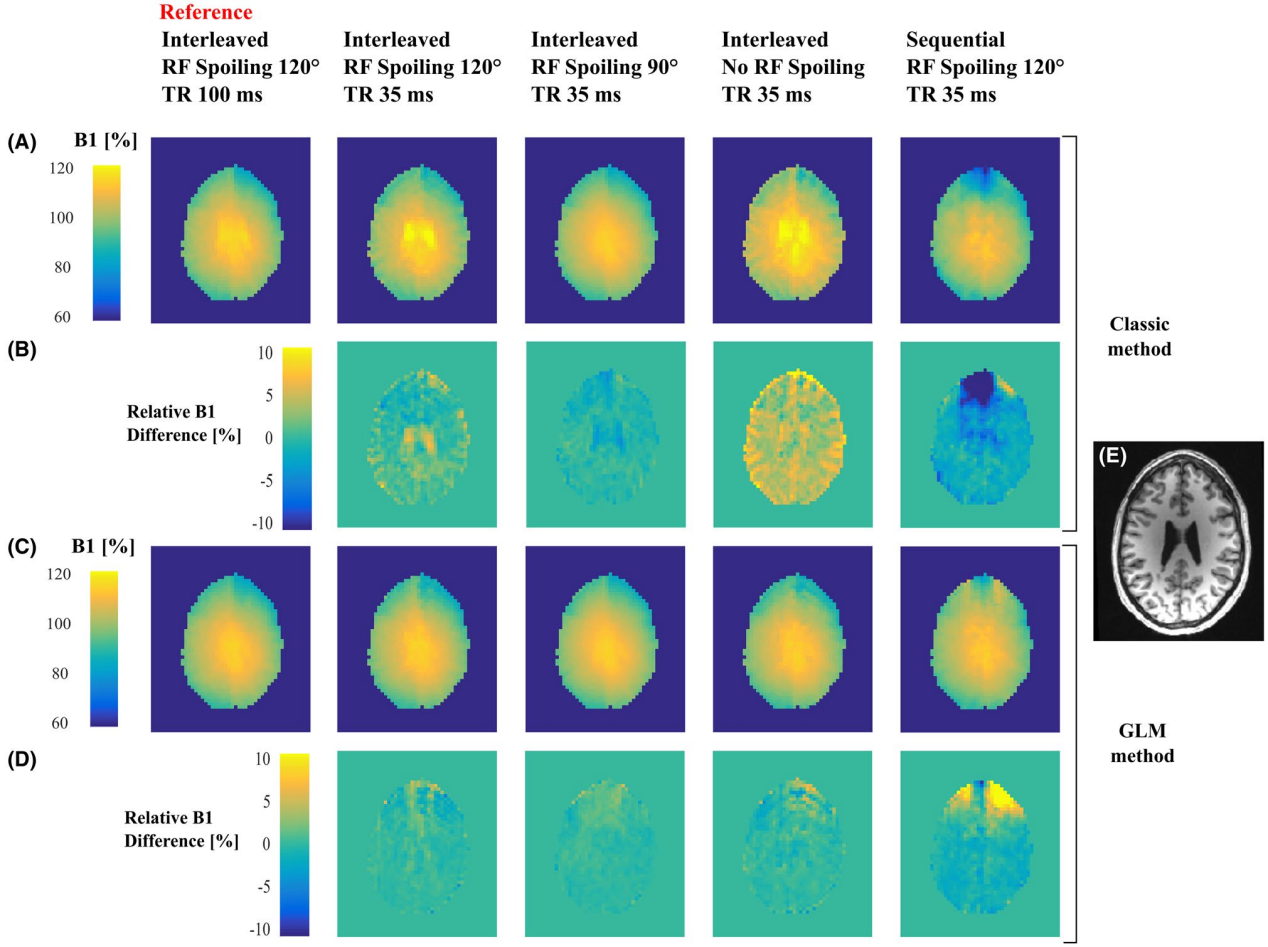
$B_{1}^{+}$ maps of one slice obtained on Participant 2 with the Classic (A) and the GLM (C) approach with 5 different protocols. Maps of the relative difference of each $B_{1}^{+}$ map with the reference maps computed with the same method: Classic (B) or GLM (D) approach. (E) Structural image of the same slice, acquired independently

**Figure 8:**
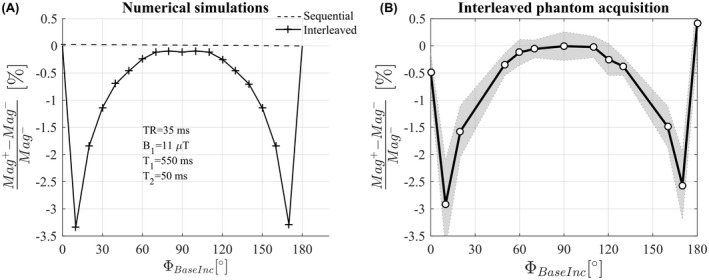
(A) Relative difference in magnitude between the 2 acquisitions with opposite off‐resonance frequencies in the case of interleaved or sequential order predicted by numerical simulations. (B) Relative difference in magnitude between the 2 interleaved acquisitions measured in phantom experiment 1

Inversion recovery and multi‐echo spin echo experiments indicate T_1_ and T_2_ times of 550 ms and 70 ms for the FBIRN phantom^23^ used. However, the latter estimation did not incorporate any correction for stimulated echoes,^31^ and the T_2_ may be as short as 50 ms. Simulations using the same sequence parameters as the phantom experiments, with a T_1_ of 550 ms, and a T_2_ of 70 ms, predicted an error of 4.9% (Figure 3D) for the case of no RF spoiling. With a shorter T_2_ of 50 ms, a lower error of 3.4% was predicted by the simulations (data not shown). These results are in broad agreement with the somewhat lower error of 3.0% observed experimentally for this case. Of note, incorporation of diffusion effects into the simulations^32^, ^33^ had little impact on the level of bias in the estimated $B_{1}^{+}$. Furthermore, while it is the phase component that is key to estimating $B_{1}^{+}$_,_ it is also worth noting that our simulations predicted a difference in the magnitude of the magnetization between TRs with interleaved off‐resonance frequencies, and that this difference would depend on the RF spoiling increment. Good agreement was again seen between prediction (see Figure 8A) and experiment (see Figure 8B). No such difference was predicted for sequential ordering of the off‐resonance frequencies. Also in agreement with the numerical simulations, the proposed GLM method removed the dependence of the $B_{1}^{+}$ estimates on the RF spoiling increment, the TR, and the acquisition mode in both phantom (Figure 5) and in vivo (Figures 6 and 7) experiments.

The robustness of the GLM method to the sequence parameters, and the RF spoiling increment in particular, makes this method more flexible, which can be exploited to optimize the signal‐to‐noise ratio. Given the dependence of the signal amplitude on the RF spoiling increment,^34^ a small gain in reproducibility can be expected by choosing the optimal value. In fact, theoretical analysis of the variance of the $B_{1}^{+}$ estimates can be used to show that the GLM should deliver higher precision. This has been verified empirically (data not shown) when using the same data for each processing method, as has been done for all of the experiments presented in this work. Although this does not affect the accuracy, it does penalize the Classic approach from a precision perspective because the TE is longer than necessary. Nonetheless, theoretical analysis would also predict improved precision with the GLM when compared with the Classic approach even with an optimal, shorter, TE. Determining the sequence settings that maximize the reproducibility and quantifying the full benefit that can be gained empirically will be the focus of future work.

In theory, the GLM method could use just a single off‐resonance frequency with a reduced model matrix containing only half‐length regressors for $X_{\mathrm{BSS}},\;X_{\omega_{B_{0}}},\;X_{Even/Odd}\;\text{and}\;X_{\mathrm{Offset}}^{+}$.^35^ In this case, $X_{\mathrm{BSS}}$ would model all the phase imparted by the BS pulse, including the component depending on B_0_ inhomogeneity and chemical shift. However, this could be corrected with the information captured by the second regressor $X_{\omega_{B_{0}}}$. However, because the BSS phase is imparted only once, the BS pulse flip angle would need to be doubled to achieve the same phase‐to‐noise ratio. This is problematic from a SAR perspective, and the benefit of a single off‐resonance frequency acquisition would be negated if the TR were also doubled to address it. Besides, removing the second acquisition with opposite off‐resonance frequency prevents the isolation of the BS phase of interest because any phase caused by the eddy currents of the crushers, for example, would also be captured by the same regressor making the problem ill‐posed. A workaround consisting of adding further gradients after the fourth echo, to distinctly capture the effects of eddy currents, has been proposed and tested but has proven to be effective only in phantom experiments.^32^

*Limitations*

Given that the GLM method relies on a multi‐echo sequence, additional pre‐processing steps are required compared with the Classic approach. Phase unwrapping across echoes is necessary. It has been necessary to spatially unwrap the phase difference between successive echoes to deal with large phase accumulation between successive echoes, then cumulatively add these to the first echo.

In the proposed method, multiple echoes are used to estimate the BSS phase, some of the echoes may suffer from dropout and potentially introduce noise into the estimate. To minimize this effect a weighted least‐squares approach has been used to estimate the parameters of the GLM, down‐weighting echoes with lower magnitude.

Conventionally, the BS pulse is applied just after the excitation pulse. Here, 2 echoes preceded the BS pulse and the difference of the third echoes, from the different off‐resonance frequency acquisitions, was used to estimate $B_{1}^{+}$ using the Classic method. This increases the minimum TE (by ~4 ms) and, therefore, lowers the signal‐to‐noise ratio relative to the single‐echo method. However, while this might reduce precision, it would not be expected to introduce bias.

This study focused on short TR 3D acquisitions. For 2D acquisitions, the shot‐to‐shot inconsistencies may be less problematic because the TR will be longer, although a bias was still observed in long T_1_ regions with a TR of 100 ms (Figure 8, first column). In addition, the 2 acquisitions of 1 slice will be more separated in time, which may result in additional phase differences due to motion, similar to the problem affecting sequential acquisitions.

Although more efficient pulses have been proposed,^18^, ^36^ only the commonly used Fermi shape for the BS pulse was investigated here. However, it can be shown that for the same imparted BS phase and the same off‐resonance frequency, the bias introduced by the interleaved acquisition order is equivalent for a Fermi pulse and the more optimized pulse design suggested by Duan et al.^18^

While we have shown how to more accurately estimate the BSS phase, the conversion to $B_{1}^{p}$ may still be a source of inaccuracy if any assumptions underlying Equation 4 are violated.^18^ For the particular conditions we have explored (a Fermi pulse with 2 ms duration, $\gamma B_{1}/\omega_{\mathrm{off}}=0.23$ and $B_{1}=11\mu T$) the error from this approximation is estimated from simulation to be less than 1%. Regardless of how the phase is converted to a $B_{1}^{+}$ value, it is imperative that the bias caused by interleaving the off‐resonance pulses be removed.

The precision and accuracy of the GLM technique and the Classic method with $\phi_{\mathrm{BaseInc}}=90^{\circ }$ relative to other $B_{1}^{+}$ mapping methods remain to be investigated. However, determining absolute accuracy will always be challenging because every method will have its own limitations.

## 6 CONCLUSIONS

Interleaved acquisitions are recommended for BS based $B_{1}^{+}$ mapping to increase robustness to motion and scanner drift. However, we have shown that, with the Classic estimation method, this can introduce error into the $B_{1}^{+}$ estimates that will depend on tissue properties and sequence settings. In theory, one could use an RF spoiling increment of 90° to be immune to this error. However, we have also proposed and validated a multi‐echo sequence design, combined with a GLM framework, to robustly isolate the BSS‐induced phase regardless of the sequence parameters used. This allows bias free, low error estimates of the $B_{1}^{+}$ efficiency that do not depend on tissue properties, sequence settings and would, furthermore, be immune to reproducible hardware imperfections. Importantly, the proposed modifications do not extend acquisition time, reduce sensitivity, or increase SAR. The latter is particularly important because SAR is a limiting factor at higher field strengths.

## Supporting information


**TABLE S1** Parameters used in the numerical simulations
