# Supplementary material for: Robust 3D Bloch‐Siegert based B1+ mapping using multi‐echo general linear modeling
Source: Magn Reson Med. 2019 Jul 18;82(6):2003–15. doi: 10.1002/mrm.27851 (PMC6771691; doi:10.1002/mrm.27851)
Supplement: Supplementary file 1 — TABLE S1 Parameters used in the numerical simulations [file MRM-82-2003-s001.docx]

S**upporting Information**

Bloch-Siegert phase shift expression

The first-order Taylor approximation of the analytical solution of the Bloch-Siegert shift is derived in this section in the same vein as (Duan et al, 2013) ^1^

In a static magnetic field, $B_{0}$, the precessional frequency is the Larmor frequency given by $\omega_{0}=\gamma B_{0}.$ The BSS approach applies an off-resonance RF pulse with a rotating frequency of $\omega_{2},$ such that $\omega_{off}=\omega_{0}-\omega_{2}\neq0.$The off-resonance pulse (termed the BS pulse) modifies the precessional frequency during the time of its application. It has an amplitude $B_{1}$ such that $\omega_{1}=\gamma B_{1}$.

In the frame of reference rotating at $\omega_{0}$, the precessional frequency during this pulse changes to:

|  | $\omega^{rot}=\sqrt{\omega_{off}^{2}+\omega_{1}^{2}}-\omega_{off}= \omega_{off}\left( \sqrt{1+\frac{\omega_{1}^{2}}{\omega_{off}^{2}}}-1 \right)$ | ( SI1.1 ) |
| --- | --- | --- |

If the off-resonance frequency of the BS pulse is much larger than its strength in frequency units, i.e. $\omega_{off}\gg\omega_{1}$, the expression reduces to:

|  | $\omega^{rot}=\frac{\omega_{1}^{2}}{2\omega_{off}}$ | ( SI1.2 ) |
| --- | --- | --- |

Given that the precessional frequency is no longer equal to $\omega_{0}$ during the BS pulse, an additional phase is accumulated. Since it is proportional to $\omega_{1}^{2}$this phase can be used to map the spatial distribution of B_1_^+^.

Allowing for local $B_{0}$ inhomogeneity, the local precessional frequency will be $\omega_{0}+\Delta\omega_{B_{0}}.$ In the rotating frame of reference and under the similar condition as before, ${(\omega}_{off}+\Delta\omega_{B_{0}})\gg\omega_{1}$, the precessional frequency $\omega^{rot}$ during the BS pulse becomes:

|  | $\omega^{rot}=\sqrt{\left( \omega_{off}+\Delta\omega_{B_{0}} \right)^{2}+\omega_{1}^{2}}- \omega_{off}$ | (SI1.3 ) |
| --- | --- | --- |
|  | $=\left( \omega_{off}+\Delta\omega_{B_{0}} \right)\sqrt{1+\frac{\omega_{1}^{2}}{\left( \omega_{off}+\Delta\omega_{B_{0}} \right)^{2}}}-\omega_{off}$ | ( SI1.4 ) |
|  | $=\frac{\omega_{1}^{2}}{2\left( \omega_{off}+\Delta\omega_{B_{0}} \right)}+\Delta\omega_{B_{0}}$ | ( SI1.5 ) |

The first term on the right hand side of this expression, representing the frequency shift due to the BS pulse, will be called $\omega_{BSS}$. Note that this effect adds to the phase accrued due to local $B_{0}$ inhomogeneity. The phase specifically accumulated due to the BS pulse, of arbitrary shape and duration T, is given by:

|  | $\phi_{BSS}=\int_{0}^{T} \omega_{BSS} dt= \int_{0}^{T} \frac{\omega_{1}^{2}}{2\left( \omega_{off}+\Delta\omega_{B_{0}} \right)}= \int_{0}^{T} \frac{\left( \gamma B_{1}\left( t \right) \right)^{2}}{2\left( \omega_{off}+\Delta\omega_{B_{0}} \right)}dt=\left( B_{1}^{p} \right)^{2}\int_{0}^{T} \frac{\left( {\gamma B}_{1}^{norm}\left( t \right) \right)^{2}}{2\left( \omega_{off}+\Delta\omega_{B_{0}} \right)} dt$ | ( SI1.6 ) |
| --- | --- | --- |

$B_{1}\left( t \right)=B_{1}^{p}*B_{1}^{norm}\left( t \right)$, where $B_{1}^{p}$ is the peak amplitude of the BS pulse, and $B_{1}^{norm}$is its normalized shape. Assuming $|\Delta\omega_{B_{0}}|\ll{|\omega}_{off}|$, the first order Taylor expansion of this expression is:

|  | $\Phi_{BSS}=\left( B_{1}^{p} \right)^{2}\int_{0}^{T} \left( {\gamma B}_{1}^{norm}\left( t \right) \right)^{2}*\left( \left[ \frac{1}{2\omega_{off}}-\frac{\Delta\omega_{B_{0}}}{2\omega_{off}^{2}} \right] +O\left( {\Delta\omega}_{B_{0}}^{2} \right) \right)dt$ | ( SI1.7 ) |
| --- | --- | --- |

1. Duan Q, van Gelderen P, Duyn J. Improved Bloch-Siegert Based B1 Mapping by Reducing Off-Resonance Shift. NMR Biomed. 2013;26:1070–1078 doi: 10.1002/nbm.2920.

Numerical simulations

Numerical simulations were carried out to evaluate the phase difference between two acquisitions with opposite off-resonance frequencies both before and after the BS pulse. A typical gradient echo acquisition was simulated in MATLAB (The MathWorks, Inc., Natick, MA) by a series of matrix operations as described below:

1. The magnetization $M=\left[ m_{x} m_{y} m_{z} \right]^{T}$ of a spin ensemble is subject to a rotation by the excitation flip angle $\alpha$ about an axis defined by the phase $\phi$ of the excitation pulse.
2. During a subsequent delay TE, longitudinal and transverse relaxation were simulated with time constants $T_{1}$and $T_{2}$, respectively. The phase at time TE is termed $\phi_{Before}^{+}$ if the off-resonance frequency of the upcoming BS pulse is positive, or $\phi_{Before}^{-}$ if the off-resonance frequency of the upcoming BS pulse is negative.
3. The application of a crusher gradient rotates the magnetization vector around the z-axis by an angle $\Omega_{1}$.
4. The BS pulse, in this work a Fermi pulse described by $B_{1}\left( t \right)=\frac{B_{1}^{p}e^{i\omega_{off}t}}{1+\exp\left( \frac{\left| t \right|-t_{0}}{a} \right)}$ , was then simulated as a series of small rotations indexed by $k\in[1:K]$. Each small rotation k is a rotation of an angle $|B_{1}\left( k*dt \right)|*dt$ about an axis defined by an azimuthal angle of $k*dt*\omega_{off}+\phi$.
5. A rotation of the resulting magnetization vector about the z-axis by an angle $-\Omega_{1}$ is then applied to simulate the crusher of opposite polarity. The resulting phase at this time is called $\phi_{After}^{+}$ if the frequency of the preceding BS pulse was positive, or $\phi_{After}^{-}$ if the frequency was negative. For simplicity, the pulse and crushers were assumed to be instantaneous from a relaxation standpoint such that $\Phi_{After}$ and $\Phi_{Before}$ have the same TE. Note that the relaxation is instead accounted for in step 7.
6. Following the second crusher gradient, a spoiler gradient was applied such that the magnetization vector from step 5 was rotated around the z-axis by an angle of $\Omega_{2}$.
7. T_1_ and T_2_ relaxation were then simulated for the remainder of the TR period, i.e. TR-TE.
8. When RF spoiling was simulated, the phase of the RF pulses, $\phi,$was incremented such that $\phi=\phi+\phi_{inc}$ and $\phi_{inc}=\phi_{inc}+\phi_{BaseInc}$.

These steps were repeated $N_{exc}$ times, by updating the magnetization of Step 1 with the magnetization vector resulting from Step 7.

The simulation was performed for a 2D grid of spin ensembles ($N_{spin}*N_{spin})$with varying values for $\Omega_{1}$ and $\Omega_{2}$ ranging from $0^{\circ}$ to $\Omega_{1}^{max}$ and $\Omega_{2}^{max}$, respectively. The values of the crusher dephasing $\Omega_{1}$ varied along one dimension of the grid, whereas the values of the spoiler dephasing $\Omega_{2}$ varied in the other direction to simulate crushers and spoiler along orthogonal axes. The integral of these spins gave the magnetisation in a single voxel.

For interleaved ordering, the sign of $\omega_{off}$ was switched before each BS pulse. For sequential ordering the sign was only switched after half the total number of pulses $\left( \frac{N_{exc}}{2} \right)$. If RF spoiling was included in the simulation, the RF spoiling phase $\left( \phi and \phi_{Inc} \right)$ were reset to 0 when the sign of $\omega_{off}$ was switched in the sequential case. In the case of an interleaved acquisition, the phase increment occurred for each repetition, including when the off-resonance frequency also changes.

The Matlab code is available here: <https://github.com/fil-physics/Publication-Code/tree/master/Bloch-Siegert>.

The numerical values of the parameters used in the simulations are listed in the Supporting Information Table S1

| $\boldsymbol{T}_{\boldsymbol{1}}$ | Longitudinal relaxation time | [550, 1350] ms |
| --- | --- | --- |
| $\boldsymbol{T}_{\boldsymbol{2}}$ | Transverse relaxation time | [70,100] ms |
| $\boldsymbol{TR}$ | Repetition time | [35, 100] ms |
| $\boldsymbol{TE}$ | Echo time for $\Phi_{Before}$ and$\phi_{After}$ | 2 ms |
| $\boldsymbol{\alpha}$ | Excitation flip angle | ${15}^{\circ}$ |
| $\boldsymbol{\omega}_{\boldsymbol{off}}$ | BS pulse off-resonance frequency | 2 kHz |
| $\boldsymbol{B}_{\boldsymbol{1}}$ | BS pulse amplitude | [8, 11] $\mu T$ |
| $\boldsymbol{T}$ | BS pulse duration | 2 ms |
| $\boldsymbol{\phi}_{\boldsymbol{BaseInc}}$ | RF spoiling increment | [0 50 117] ° (Figure 2)  [0:10:180] ° (Figure 3) |
| $\boldsymbol{\Omega}_{\boldsymbol{1}}^{\boldsymbol{max}}$ | Crusher gradient dephasing moment | $8 \pi$ |
| $\boldsymbol{\Omega}_{\boldsymbol{2}}^{\boldsymbol{max}}$ | Spoiler gradient dephasing moment | $4 \pi$ |
| $\boldsymbol{N}_{\boldsymbol{spin}}$ | Number of spins in ensemble along each dimension | 100 |
| $\boldsymbol{N}_{\boldsymbol{exc}}$ | Number of excitation pulses simulated | 300 |
| $\boldsymbol{a}$ | Fermi pulse parameter | 0.16 ms |
| $\boldsymbol{t}_{\boldsymbol{0}}$ | Fermi pulse parameter | 3 ms |

Supporting Information Table S1: Parameters used in the numerical simulations

The BS phase estimated by the Classic approach is simulated as:

| $\Phi_{BSS}^{Classic}=\frac{{\Phi_{After}^{+}-\Phi}_{After}^{-}}{2}$ | ( SI2.1) |
| --- | --- |

The BS phase estimated by the GLM approach is simulated as:

| $\Phi_{BSS}^{GLM}=\frac{\left( {\Phi_{After}^{+}-\Phi}_{Before}^{+} \right)-\left( {\Phi_{After}^{-}-\Phi}_{Before}^{-} \right)}{2}$ | ( SI2.2) |
| --- | --- |
